# The Transcription Factor PoCon7 Is Essential for Fungal Viability and Regulates Chitinase Gene Expression in *Penicillium oxalicum*

**DOI:** 10.3390/ijms27010333

**Published:** 2025-12-28

**Authors:** Kexuan Ma, Haiyi Yuan, Jian Zhao, Yuqi Qin

**Affiliations:** 1 National Glycoengineering Research Center, Shandong University, Qingdao 266237, China; makexuan@mail.sdu.edu.cn (K.M.); yuanhaiyi@mail.sdu.edu.cn (H.Y.); 2 State Key Laboratory of Microbial Technology, Shandong University, Qingdao 266237, China; zhaojian@sdu.edu.cn

**Keywords:** chitinases, fungi, regulation, Snf1, transcription factors

## Abstract

The dynamic remodeling of the fungal cell wall depends on a balance between chitin synthesis and degradation. Chitinases are critical for nutrient acquisition, cell wall remodeling, and defense; yet, the upstream regulatory mechanisms controlling chitinase gene expression remain poorly understood. Here, Tandem Affinity Purification–Mass Spectrometry (TAP–MS) with the *Penicillium oxalicum* Snf1 kinase (*Po*Snf1) as bait identified the zinc finger transcription factor (TF) *Po*Con7 as a putative target of the Snf1 kinase complex. This complex comprises the catalytic α subunit Snf1, one of three alternative β subunits Gal83, and the γ subunit Snf4. Although *Po*Con7 does not directly bind *Po*Snf1 or *Po*Snf4, it specifically interacts with *Po*Gal83. Phylogenetic analysis indicates that *Po*Con7 is a conserved, nuclear-localized C2H2-type TF in filamentous fungi. *Po*Con7 is likely essential for fungal viability, as only a truncated mutant (con7-B) could be generated, while full deletion was lethal. The con7-B mutant displayed delayed hyphal extension, reduced conidiation, downregulation of developmental genes, and upregulation of cell wall-degrading enzyme (CWDE) genes. DNA Affinity Purification Sequencing (DAP-seq) revealed that *Po*Con7 binds target gene promoters via the motif 5′-TATTWTTAT-3′. ChIP-qPCR confirmed *Po*Con7 enrichment at specific sites within the chitinase genes *chi18A* and *chi18C*, and the disruption of *Po*Con7 markedly reduced their expression. Thus, *Po*Con7 represents the first TF shown to directly regulate chitinase gene expression in filamentous fungi.

## 1. Introduction

The fungal cell wall, which serves as the primary interface with the external environment, is a dynamic structure essential for maintaining cell integrity, morphology, and interaction with surroundings. Its composition is predominantly polysaccharide-based, with glucans and chitin accounting for approximately 90% of the cell wall’s dry weight [1]. Chitin is the second most abundant natural polymer after cellulose and is a key structural component in fungal cell walls and invertebrate exoskeletons [2,3]. The dynamic remodeling of the cell wall in these fungi relies on the coordinated actions of chitin synthases and chitinases [4,5].

Chitinases (EC 3.2.1.14) are hydrolytic enzymes that cleave β-1,4-glycosidic bonds in chitin and chito-oligosaccharides [6]. In fungi, chitinases fall exclusively within the glycoside hydrolase family 18 (GH18) [7,8]. Functionally, fungal chitinases participate in a range of physiological processes: (i) the degradation of exogenous chitin from fungal hyphae or arthropod exoskeletons for nutrient acquisition [9,10]; (ii) involvement in cell wall remodeling during hyphal elongation, branching, fusion, and autolysis [11,12]; and (iii) defense against competing fungi or predators [13]. This functional diversity implies the existence of sophisticated regulatory mechanisms to ensure precise control of chitinase expression. While some chitinase genes are known to be induced by chitin or cell wall components [14], the upstream regulators and associated signaling pathways remain largely unidentified.

One conserved eukaryotic kinase complex implicated in metabolic and developmental regulation is SNF1/AMPK, which in fungi comprises a catalytic α-subunit (Snf1), a γ-subunit (Snf4), and one of three alternative β-subunits (Gal83, Sip1, or Sip2) [15]. This complex is a key mediator of carbon catabolite repression and energy sensing [16,17]. It has been reported that the Snf1 kinase complex targets multiple transcription factors (TFs), such as Mig1, Adr1, Cat8, and Sip4 in *Saccharomyces cerevisiae* [18]. In several filamentous fungi, Snf1 orthologs have been shown to influence chitinase gene expression and cell wall integrity. For example, the deletion of SNF1 in *Cordyceps militaris*, *Metarhizium acridum*, and *Beauveria bassiana* led to a significant downregulation of chitinase genes and impaired cell wall remodeling [19,20,21].

*Penicillium* spp. are well-known fungi that play important roles in biotechnology and in the medical and food industries [22]. *Penicillium oxalicum* is an excellent secretor of cell wall degrading enzymes (CWDEs) and represents a promising microbial platform for CWDE production in white biotechnology [23,24]. The chitinase Chi18A, one of the top ten secreted proteins, is co-secreted with CWDEs [24]. Studies in *Penicillium digitatum* have shown that chitin metabolism not only regulate hyphal morphology and chitin content, but also significantly affect conidiation and pathogenicity [25]. Increased activities of chitinases and glucanases are often accompanied by a notable reduction in chitin and glucan content within the cell wall [26], indicating a close relationship between chitinase activity and cell wall composition. Moreover, normal growth and development in *Penicillium marneffei* are closely linked to cell wall structural integrity [27].

In this study, a homolog of TF Con7 was identified as a putative target of the Snf1 kinase complex in *P. oxalicum*. Functional characterization confirmed its essential role in fungal viability, hyphal growth, and conidiation. *Po*Con7 directly binds to chitinase gene promoters to regulate their expression, establishing *Po*Con7 as their direct transcriptional regulator.

## 2. Results

### 2.1. TAP-MS Identifies PoCon7 as a Novel Component Associated with the Snf1 Complex

Multiple studies have indicated that Snf1 orthologs play a critical role in chitinase expression and/or cell wall remodeling [19,20,21]. To elucidate the underlying mechanism, we sought to identify the key target proteins through which Snf1 mediates these functions. We used Tandem Affinity Purification coupled with Mass Spectrometry (TAP–MS), a method highly effective in identifying in vivo protein partners of a protein of interest in fungi [28,29,30], as the two-step purification process can effectively reduce proteins that bind non-specifically. The Snf1-TAP strain was constructed by attaching the HA-FLAG tag to the C-terminus of *Po*Snf1 (PDE_02007, UniProt Entry S7ZEF0). The native *Po*Snf1 in the *P. oxalicum* wild-type (WT) strain 114-2 was used as a control, with two biological replicates for the TAP-MS experiment. After the two-step tandem purification, the final eluate was divided into three sections: one designated for SDS-PAGE, another for Western blot, and the third for MS-MS assay to identify the potential interacting proteins of the *Po*Snf1 bait.

**Figure 1:**
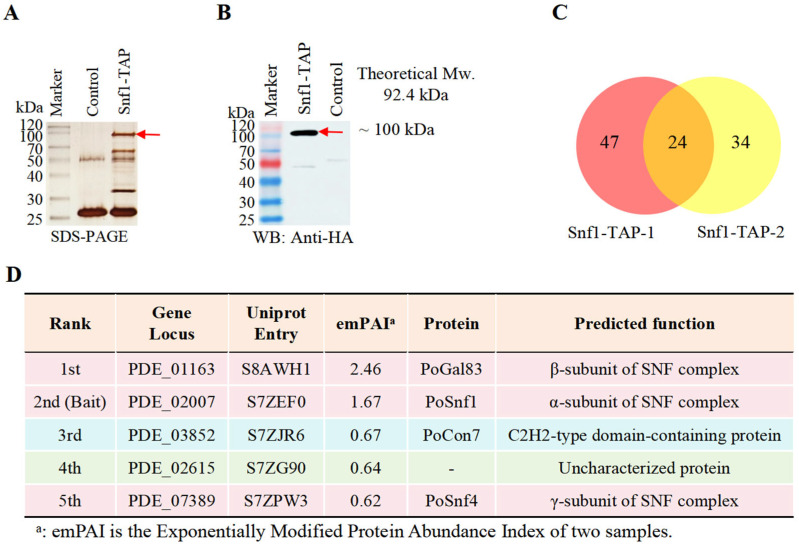
Results from TAP-MS with *Po*Snf1 as the bait. (**A**) Silver staining after SDS-PAGE of Snf1-TAP eluate. (**B**) Western blot of Snf1-TAP eluate using an anti-HA antibody. (**C**) Overlapping proteins in two Snf1-TAP-1 and Snf1-TAP-2 biological samples. (**D**) The top 5 proteins identified through TAP-MS. Light blue: bait protein *Po*Con7; light pink background: three subunits of the Snf1 kinase complex; light green background: other proteins. Detailed information, including Unique_PepCount identified by LC-MS/MS, gene locus, theoretical PepCount, emPAI, and predicted functions of potential interacting proteins, is available in Supplementary Spreadsheet S1.

*Po*Snf1 protein is theoretically 92.4 kDa in molecular weight. An ~100 kDa band was detected between the control (WT) and Snf1-TAP strains on the SDS-PAGE gel (Figure 1A, red arrow), as confirmed by Western blot analysis (Figure 1B, red arrow).

To identify the *Po*Snf1 bait and its possible interacting proteins, the third section of the eluent was examined using LC-MS/MS. The proteins identified in the two biological replicates, Snf1-TAP-1 and Snf1-TAP-2, via TAP-MS are detailed in Supplementary Spreadsheet S1. Based on the exponentially modified protein abundance index (emPAI) [31], the identified proteins were ranked to estimate their abundance within the sample. The samples Snf1-TAP-1 and Snf1-TAP-2 contained 71 and 58 identified proteins, respectively. Credible proteins that may interact with *Po*Snf1 include 24 proteins identified in two Snf1-TAP samples but not in any control samples (Figure 1C and Supplementary Spreadsheet S1). In Figure 1D, the five proteins with the highest emPAI rankings are presented.

The bait *Po*Snf1 is ranked second as the α subunit of Snf1 kinase complex.

The protein with the highest emPAI is PDE_01163 (Uniprot Entry S8AWH1). We name PDE_01163 as *Po*Gal83 because its homolog in *S. cerevisiae* is protein Gal83, which is the β subunit of the Snf1 kinase complex that functions as an adaptor that brings Snf1 and Snf4 into proximity and also contributes to the substrate specificity of the kinase complex [32].

The protein with the third-highest emPAI is PDE_03852 (Uniprot Entry S7ZJR6), a C2H2-type domain-containing protein. PDE_03852 was named *Po*Con7 based on its homology to the Con7p TF in *Magnaporthe oryzae* and *F. graminearumis* is Con7 [33,34].

The protein ranked 4th (PDE_02615) is an uncharacterized protein.

The protein ranked 5th (PDE_07389) is named *Po*Snf4 because its homolog in *S. cerevisiae* is Snf4, the γ subunit of the Snf1 complex that binds to and activates the kinase catalytic subunit under stress conditions [35].

In summary, all three subunits of the Snf1 kinase complex, including the α-subunit (Snf1), β-subunit (Gal83), and γ-subunit (Snf4), were detected in the TAP-MS results, confirming the integrity of the complex. Additionally, a novel interacting protein, *Po*Con7, was identified, suggesting its identification as a potential target of the Snf1 kinase complex.

### 2.2. PoCon7 Interacts Directly with PoGal83

**Figure 2:**
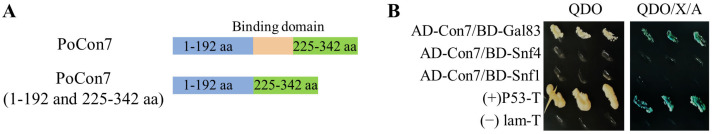
The results of Y2H. (**A**) Strategy of Y2H. DNA-binding domain (193–224) was removed from *Po*Con7 as the intact protein showed autoactivation. (**B**) Hybridization results of Y2H Gold-BD-*Po*Gal83/Snf4/Snf1 and Y187-AD-*Po*Con7 strains on QDO (quadruple-dropout, SD-Ade/-His/-Leu/-Trp) and QDO/x-α-gal/Aba (QDO supplemented with X-α-gal and aureobasidin A).

Given that the TAP results may include indirect interactions mediated by bridging proteins, a yeast two-hybrid (Y2H) assay was performed to determine: (1) whether *Po*Con7 interacts directly with the *Po*SNF complex; and (2) which subunit or subunits of the complex are responsible for this interaction. Y2H strains were constructed to express *Po*Con7 along with each individual subunit of the Snf1 kinase complex *Po*Snf1, *Po*Snf4, and *Po*Gal83. After confirming the absence of toxicity and autoactivation in these strains (Supplementary Figure S1), direct interaction was observed specifically between *Po*Con7 and *Po*Gal83. No direct interaction was detected between *Po*Con7 and either *Po*Snf1 or *Po*Snf4 (Figure 2).

### 2.3. PoCon7 Is a Conserved Nuclear-Localized TF in Filamentous Fungi

To further characterize *Po*Con7, we performed phylogenetic analysis and subcellular localization determination.

**Figure 3:**
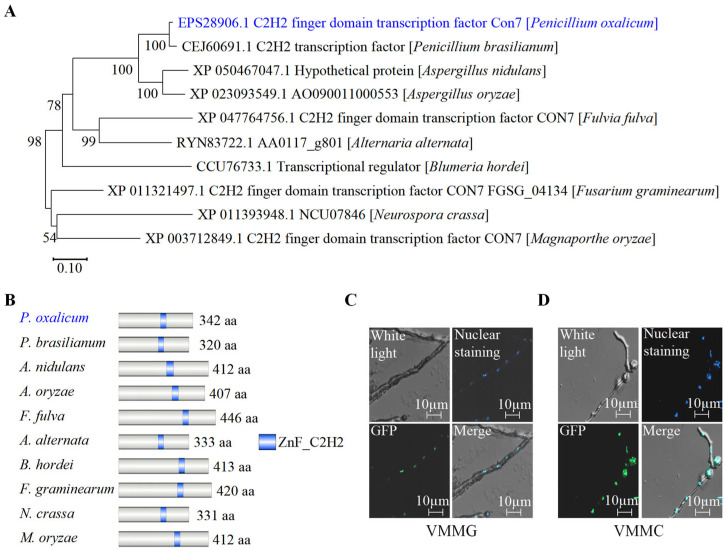
Analysis of phylogenetic relationships and subcellular localization of *Po*Con7. (**A**) Construction of the phylogenetic tree of the *po*con7 homologous protein. (**B**) Domain analysis of *Po*Con7 homologous protein in different filamentous fungi. Numbers indicate the total count of amino acids for each protein. For example, 342 aa indicates that there are 342 amino acids. The conserved C2H2 zinc-finger domains are highlighted in blue. These figures were made after the corresponding protein sequence was scaled in a uniform scale. (**C**) The subcellular localization of *Po*Con7 on VMMG. (**D**) The subcellular localization of *Po*Con7 on VMMC. This picture has four parts: upper left, white light; upper right, Hoechst 33,342 was used to stain nuclei in blue; bottom left, green fluorescence; bottom right, merged image of green fluorescence and nuclear staining.

Using the protein sequence of *Po*Con7, a BLASTP search was performed on the landmark database (http://blast.ncbi.nlm.nih.gov/smartblast/smartBlast.cgi?) (accessed on 10 June 2024), which comprises 27 genomes from well-researched reference species covering a diverse taxonomic range. In well-known model organisms like *Escherichia coli*, *Bacillus subtilis*, *S. cerevisiae*, *Schizosaccharomyces pombe*, *Caenorhabditis elegans*, *Mus musculus*, and *Drosophila melanogaster*, no homologous protein exists. *Po*Con7, despite not being identified in well-researched reference species, is phylogenetically conserved and present in different filamentous fungi (Figure 3A and Supplementary Spreadsheet S2). The study of domain architectures in several Con7 orthologs from different filamentous fungi indicated that each contains a C2H2 domain (Figure 3B).

To assess its subcellular localization, we generated a Con7-GFP strain in *P. oxalicum* by replacing the native *Po*Con7 with a GFP-tagged version. Since the nuclear localization of TFs can be signal-dependent [36], we examined the localization of *Po*Con7 under two different carbon source conditions: Vogel’s minimal medium (VMM) supplemented with 2% glucose (VMMG) or 2% cellulose (VMMC). VMMG, with glucose as the carbon source, mimics nutrient-rich environments commonly used in laboratory or industrial fermentation settings, where readily metabolizable sugars are available. In contrast, VMMC, containing cellulose as the sole carbon source, simulates the natural ecological niche of fungi, where they commonly encounter plant cell walls in which cellulose serves as the primary structural polymer. An overlap of green fluorescence (Figure 3C,D, bottom left) and nuclear staining (Figure 3C,D, top right) was observed on the merged image (Figure 3C,D, bottom right), regardless of whether strain Con7-GFP was cultured on VMMG or VMMC. The results indicate that *Po*con7 is localized in the nucleus, whether under glucose or cellulose signal.

### 2.4. PoCon7 Is Essential for Fungal Viability and Is Required for Normal Growth and Conidiation

**Figure 4:**
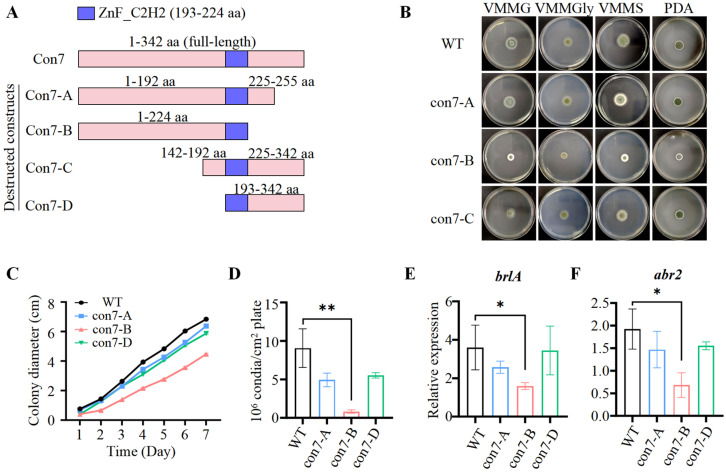
Colony morphology and conidiation of WT and *Po*Con7-associated mutants. (**A**) Schematic illustration of *Po*Con7-associated mutants. (**B**) The colony morphology of strains cultivated on VMMG, VMMGly, and VMMS agar or PDA. The strains were cultivated on at 30 °C for 4 days. (**C**) Colony diameters on VMMG agar. (**D**) Levels of conidiation of the colony in 4-day-old cultures on VMMG agar. Assay of the transcription levels of genes *brlA* (**E**) and *abr2* (**F**). Gene expression copy numbers were calculated using the standard curves constructed for each gene, and the data were then normalized with the expression levels of the actin gene. Three biological triplicates were performed, and the mean values and standard deviations were calculated. Statistical analysis was performed with a one-tailed homoscedastic (equal variance) *t*-test. * *p* < 0.05, ** *p* < 0.01.

To further elucidate the biological role of *Po*Con7, we first attempted to generate a complete deletion mutant. However, this effort was unsuccessful. We hypothesized that *Po*Con7 deletion might be lethal in *P. oxalicum*. Then, we adopted alternative strategies to construct disruptive mutants of *Po*Con7. Four mutant designs (A–D) were engineered (Figure 4A), three of which (con7-A, con7-B, and con7-C) were successfully generated.

On solid media including VMMG, VMMGly (VMM with 2% glycerol), VMMS (VMM with 2% sucrose), and potato dextrose agar (PDA), the con7-A and con7-C mutants showed no significant differences in growth or colony morphology compared to the WT. The con7-B mutant, however, exhibited not only reduced radical growth but also a significantly lighter colony color than the WT (Figure 4B). Taking its growth on VMMG agar as an example: the colony diameter of con7-B was only 57.2% of that of the WT after 5 days of cultivation (Figure 4C). Interestingly, when cultured in VMMG liquid medium, the con7-B mutant did not show a significant difference in biomass compared to the WT (Supplementary Figure S2). This is likely because the smaller colony size on solid media reflects impaired polarized hyphal extension necessary for surface colonization, whereas the unchanged biomass in liquid culture indicates that the mutation does not affect the overall metabolic capacity for proliferation.

The lighter colony color of the con7-B mutant could be attributed to reduced conidiation and/or impaired spore pigment synthesis. Measurement of conidial production confirmed a severe conidiation defect, with the con7-B mutant yielding only ~9.0% of the conidia produced by the WT (Figure 4D). We then investigated the expression of key regulatory genes of conidiation. In *P. oxalicum*, a central regulatory pathway for asexual development consists of three TFs (BrlA → AbaA → WetA), among which BrlA is the most critical and is necessary to drive conidiation [37,38]. RT-qPCR analysis revealed that *brlA* transcription was significantly downregulated in the con7-B mutant (Figure 4E). In addition, given that pigments are essential structural components of the spore wall and that *P. oxalicum* possesses a predicted dihydroxynaphthalene (DHN)–melanin biosynthesis pathway (Abr2 → Abr1 → Ayg1 → Arp1 → Arp2 → PksP/Alb1) [39,40], we analyzed the expression of *abr2*, the first gene in this pathway. The results showed that *abr*2 expression was also markedly reduced in the con7-B mutant (Figure 4F).

### 2.5. PoCon7 Directly Binds and Regulates Chitinase Genes

**Figure 5:**
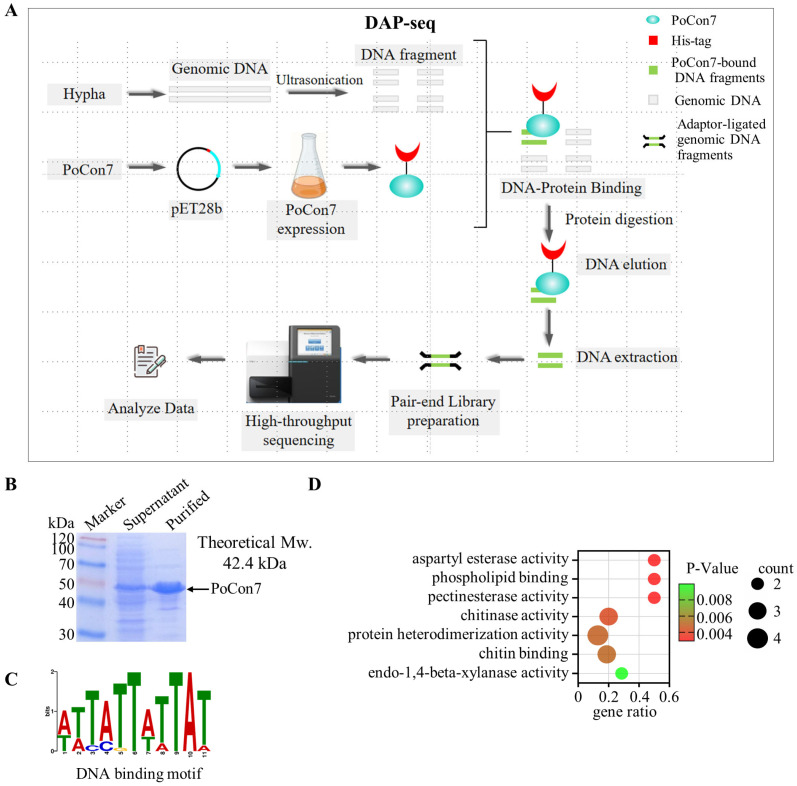
The DAP-seq experiment of *Po*Con7. (**A**) Schematic workflow diagram of DAP-seq. (**B**) SDS-PAGE analysis of *Po*Con7 recombinantly expressed in *E. coli*. (**C**) DNA binding motif enrichment analysis of *Po*Con7. (**D**) GO enrichment of peak-related genes in Con7.

To elucidate the molecular basis of the lethal phenotype caused by the absence of *Po*Con7, we sought to identify its direct genomic targets. DNA Affinity Purification sequencing (DAP-seq) was performed to map *Po*Con7-binding sites across the genome (Figure 5A). *Po*Con7 was expressed in *E. coli* (Figure 5B), and then the purified recombinant *Po*Con7 was subjected to DAP-seq analysis, which identified 211 high-confidence peak regions located within promoter sequences (Supplementary Spreadsheet S3). The binding motif of *Po*Con7 was verified as 5′-TATTWTTAT-3′ (Figure 5C). GO enrichment analysis revealed that 211 peaks were significantly enriched in terms including “chitinase activity” and “chitin binding” (Figure 5D and Supplementary Spreadsheet S4). These enrichment results demonstrate that *Po*Con7 regulates genes related to chitinases.

### 2.6. ChIP-qPCR Reveals the Enrichment of PoCon7 at Promoter Regions of Key Chitinase Genes

**Figure 6:**
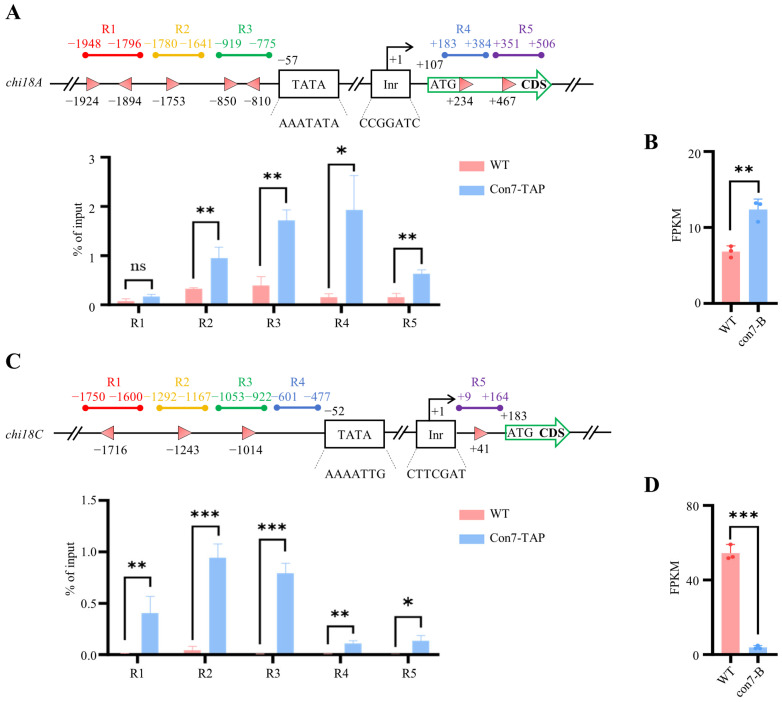
*Po*Con7 enrichment in the specific regions of chitinase genes assayed by ChIP-qPCR and effects of *Po*Con7 on the transcription levels of chitinase genes when the WT strain is cultivated on VMMG agar. (**A**) Chitinase gene *chi18A*. (**B**) The expression level of *chi18A* in con7-B mutant. (**C**) Chitinase gene *chi18C*. (**D**) The expression level of *chi18C* in con7-B mutant. Top subgraphs depict ChIP-qPCR strategies employed for each gene, with the transcription start site (TSS) positioned at +1. The initiator (Inr) and TATA box elements are illustrated. Pink triangles denote *Po*Con7 DNA-binding sites, with their orientation indicating the orientation of the binding motif. Bottom subgraphs present ChIP-qPCR results, with relative enrichment of IP DNA calculated as a percentage of input. All data represent average values obtained from measurements in biological triplicates, with error bars denoting standard deviations. Statistical significance is indicated as follows: * *p* < 0.05, ** *p* < 0.01, and *** *p* < 0.001. FPKM stands for fragments per kilobase of transcript per million mapped fragments.

To further validate the binding status of *Po*Con7 to its target genes, we performed ChIP-qPCR assays. The target genes selected were *chi18A* (PDE_08122) and chi18C (PDE_06566). *chi18A* was the most abundant secreted chitinase in *P. oxalicum* [41], while PDE_06566 was identified as a chitinase in DAP-seq screening. ChIP-qPCR methods and results appear in Figure 6 and Supplementary Spreadsheet S5. Five representative regions (regions 1–5) encompassing upstream sequences and CDS were analyzed for each gene.

When designing the ChIP-qPCR assay for *chi18A*, we positioned regions 1, 2, and 3 (R1/2/3) approximately 2000 bp upstream (-) of the transcription start site (TSS), while region 4 (R4) and region 5 (R5) were positioned near the 5′ region of CDS. The ChIP-qPCR results reveal that *Po*Con7 binds to regions 2/3/4 and the 5′-CDS in the Con7-TAP strain. Notably, regions 3 and 4 show significantly higher enrichment levels compared to regions 2 and 5. However, despite containing two *Po*Con7 binding sites, region 1 exhibits no substantial enrichment (Figure 6A).

The ChIP-qPCR design of the *chi18C* gene is similar to that of the *chi18A* gene. All four regions (R1/2/3/4) are situated approximately 2000 bp upstream (-) of the TSS, while region 5 (R5) is positioned near the 5′ region of CDS. Notably, *Po*Con7 enrichment was detected across every region examined (R1/2/3/4/5), with the most pronounced enrichment occurring at R2 and R3. These findings suggest that *Po*Con7 directly interacts with chitinase genes (Figure 6C). Moreover, transcriptome data reveal significant changes in the expression levels of *chi18A* and *chi18C* (Figure 6B,D), further supporting that con7 directly regulates the expression of chitinase genes.

### 2.7. Transcriptomic Profiling Reveals an Extensive Regulatory Role for PoCon7

To analyze the global regulatory role of *Po*Con7, we further performed transcriptome sequencing (RNA-seq) analyses on the WT and con7-B mutant cultured in VMMG and VMMC liquid medium.

**Figure 7:**
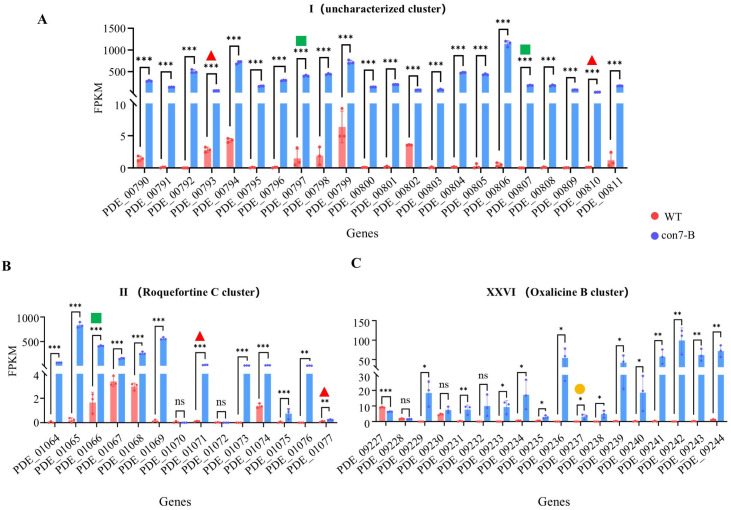
Effects of *Po*Con7 on regulating secondary metabolic clusters. Expression patterns of secondary metabolite biosynthetic gene clusters I (**A**), II (**B**), and XXVI (**C**). Core backbone biosynthetic genes in each BGC are denoted: red solid triangle: nonribosomal peptide synthetase (NRPS) gene; green solid square: dimethylallyltryptophan synthase (DMATS) gene; orange solid circle, polyketide synthase (PKS) gene. All data represent average values obtained from measurements in biological triplicates, with error bars denoting standard deviations. Statistical significance is indicated as follows: * *p* < 0.05, ** *p* < 0.01, and *** *p* < 0.001. FPKM stands for fragments per kilobase of transcript per million mapped fragments.

Under glucose condition, compared to the WT strain, the con7-B mutant exhibited 1856 differentially expressed genes (DEGs) (|log_2_(FoldChange)| ≥ 1, Q-value < 0.05), with 1190 upregulated and 666 downregulated (Supplementary Spreadsheet S6). GO enrichment analysis of upregulated genes in the con7-B mutant revealed associations with biological processes (BPs) related to the growth and development of fungi, including “Phenol-containing compound metabolic process”, “Phenol-containing compound biosynthetic process”, and “Terpenoid/Isoprenoid metabolic process” (Figure S3). These findings suggest that *Po*Con7 might regulate secondary metabolism. There are 28 predicted secondary metabolite biosynthetic gene clusters (BGCs) in *P. oxalicum* (Supplementary Spreadsheet S7) [42]. We analyzed 28 clusters and noticed these three clusters. All genes in Cluster I (PDE_00790—PDE_00811) were significantly upregulated (Figure 7A), though the secondary metabolite encoded by this cluster remains unknown. Nearly all genes in Cluster II (PDE_01064—PDE_01077) exhibited significantly upregulation (Figure 7B), and this cluster is responsible for the production of the secondary metabolite roquefortine C [43]. Similarly, Cluster XXVI (PDE_09227—PDE_09244) also showed an upregulation of almost all genes (Figure 7C), which has been reported to encode the biosynthesis of oxalicine B [44]. However, the DAP-seq data did not identify significant peaks in the promoters of the core backbone genes of these clusters. The result suggests that *Po*Con7 does not directly regulate these backbone genes by binding to their promoters. The results indicate that Con7 affects the synthesis of secondary metabolites indirectly.

Under cellulose condition, compared to the wild-type (WT) strain, the con7-B mutant exhibited 1,219 differentially expressed genes (DEGs) (|log_2_(FoldChange)| ≥ 1, Q-value < 0.05), with 620 upregulated and 599 downregulated (Supplementary Spreadsheet S8). GO enrichment analysis of the upregulated genes in the con7-B mutant revealed associations with biological processes (BPs) related to cell wall component metabolism, including “Polysaccharide metabolic process”, “Carbohydrate metabolic process”, and “Cellulose catabolic process” (Supplementary Figure S3). Notably, polysaccharides such as cellulose and chitin are key structural components of plant and fungal cell walls. The enriched cellular component (CC) terms included “extracellular region” and “plasma membrane” (Supplementary Figure S3). The extracellular region is exactly the localization where cell wall-degrading enzymes (CWDEs) are secreted and enriched. Molecular function (MF) terms included “hydrolase activity, hydrolyzing O-glycosyl compounds”, where a major component of O-glycosyl compounds is cell wall polysaccharides, represented by cellulose, hemicellulose, and pectin (Supplementary Figure S3). These findings collectively suggest that *Po*Con7 disruption may promote the upregulation of CWDE-encoding genes. Furthermore, we examined the intersection between the DEGs (|log_2_(FoldChange)| ≥ 1, Q-value < 0.05) from transcriptomes and the 211 DAP-seq genes. This intersection identified 56 genes, among which was the chitinase gene *chi18C* (Supplementary Spreadsheet S9), providing evidence for the direct regulation of chitinase genes by *Po*Con7.

### 2.8. Disruption of PoCon7 Perturbs the Transcriptional Regulatory Network to Indirectly Enhance (Hemi)Cellulase Expression

**Figure 8:**
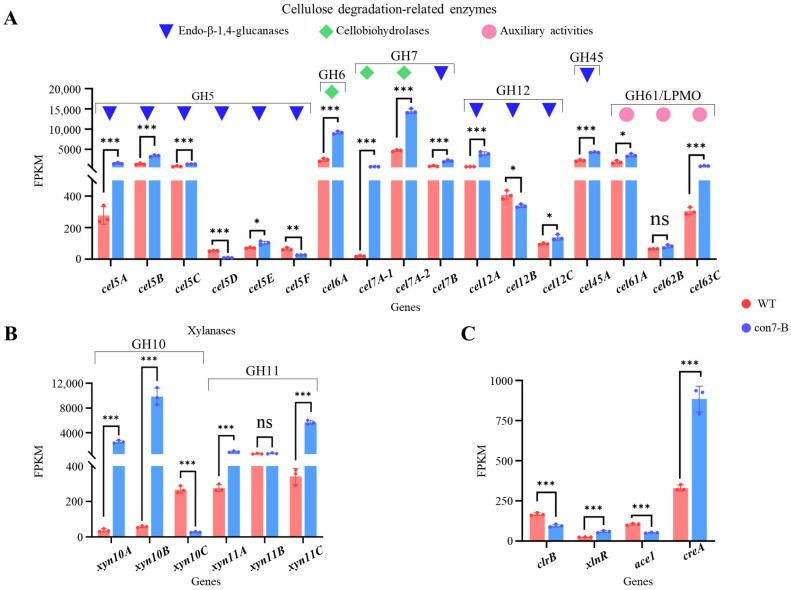
Effects of *Po*Con7 on transcription levels of genes encoding key CWDEs and TFs. Expression patterns of cellulose degradation-related genes (**A**), xylanase genes (**B**), and key TF genes (**C**). FPKM, fragments per kilobase of transcript per million mapped fragments. Statistical significance is indicated as follows: * *p* < 0.05, ** *p* < 0.01, and *** *p* < 0.001.

Then, we analyzed the expression of genes encoding the major cellulose-degrading enzymes among the CWDEs. Among the 17 cellulose-degrading enzymes, comprising 14 cellulases (11 endo-β-1,4-glucanases and 3 cellobiohydrolases) classified into glycoside hydrolase (GH) families 5, 6, 7, 12, and 45, and 3 lytic polysaccharide monooxygenases (LPMOs; formerly GH61), 14 genes were upregulated in the con7-B mutant (Figure 8A). Notably, the expression levels of the two most abundantly secreted endoglucanases (Cel7B/EG1 and Cel5A/EG2) and the two major cellobiohydrolases (Cel7A-2/CBH1 and Cel6A/CBH2) were all significantly upregulated. Furthermore, expression analysis of the six most abundantly secreted xylanases (three from GH10 and three from GH11 families) revealed that four of the corresponding genes were significantly upregulated in the con7-B mutant (Figure 8B).

We also analyzed the expression of four key TFs known to regulate (hemi)cellulase expression: the negative regulators Ace1 and CreA [30,45], and the positive regulators ClrB and XlnR [46,47]. Transcriptome analysis revealed that in the con7-B mutant, the expression of the transcriptional repressor CreA and the activator XlnR was upregulated, whereas the activator ClrB and the repressor Ace1 were downregulated (Figure 8C). However, the DAP-seq data did not identify significant peaks in the promoters of the TF genes *clrB*, *xlnR*, *ace1*, or *creA*. The result suggests that *Po*Con7 does not directly regulate these TFs by binding to their promoters. Therefore, the enhancement of the (hemi)cellulase gene expression occurs through an indirect mechanism. These alterations suggest that *Po*Con7 likely perturbs the transcriptional regulatory network, triggering a cascade of effects that indirectly drive the upregulation of (hemi)cellulase genes expression.

## 3. Discussion

Our study demonstrates that the TF *Po*Con7, a downstream target of the Snf1 complex, is likely essential for fungal viability and functions as a direct upstream regulator of chitinase genes.

This finding is similar to the results observed for its homologous proteins, *Mo*Con7 in *M. oryzae* and *Fg*Con7 in *F. graminearum*. In the rice blast fungus *M. oryzae*, TF MoCon7 was first identified and found to affect pathogenicity and cell wall composition [33]. The study on *F. graminearum* demonstrates that *Fg*Con7 is essential for conidiation as it regulates master regulator genes of conidiation [34]. Nevertheless, the upstream regulators of Con7, the genes it directly regulates, and the DNA motifs to which it binds have not been fully elucidated.

In *P. oxalicum*, the complete deletion of the *Pocon7* gene could not be obtained, indicating that it is likely essential for fungal viability. Similarly, studies on *M. oryzae* relied on the partial disruption of *Mocon7* via T-DNA insertion in the promoter region that severely reduces its expression, rather than a full knockout [33]. While we propose that Con7’s role in regulating chitinases may influence growth, the individual deletion of chitinase genes typically does not cause cell death in most fungi [48,49,50]. This is likely due to functional redundancy within the gene family [51]. Therefore, we speculate that the deletion lethal phenotype produced by *Po*Con7 as an essential gene may stem from two mechanisms. First, *Po*Con7 may directly bind to and coregulate multiple chitinase genes, and its absence could severely disrupt normal fungal growth. Second, based on the DAP-seq results, *Po*Con7 was found to bind to the promoter regions of multiple other genes involved in stress response, signal transduction, and regulation, for example, PDE_03760 and PDE_09149. Its homologous protein in *S. cerevisiae* is Hsp70 and Hsp90. The conserved Hsp90 and Hsp70 molecular chaperones are essential for proteome maintenance [52]. Maintaining a healthy proteome is fundamental for the survival of all organisms [53].

*Po*Con7 binds to and regulates chitinase genes. This finding aligns with the established role of its ortholog, *Mo*Con7, in *M. oryzae*. There, *Mo*Con7 is a master regulator of cell wall homeostasis, and its disruption leads to the dysregulation of multiple genes involved in chitin synthesis, binding, and modification [33]. Indeed, associations between TFs and chitinase regulation have been noted in other fungi. For example, in *Aspergillus nidulans*, both TF RlmA and RlmA-independent factors regulated the expression of chitinase genes *chiA* and *chiB* [54]. In *Trichoderma reesei*, the deletion of TF gene *xyr1* led to the upregulation of chitinase-encoding genes [55]. Similarly, in *M. oryzae*, the suppression of the TF OsNAC111 resulted in a reduced expression of chitinase genes [56]. However, none of these TFs have been established as directly binding to and regulating chitinase genes. Moreover, the con7-B mutant exhibits growth and developmental defects (Figure 4). We propose that these defects are closely linked to the biological role of *Po*Con7 as a transcriptional regulator of chitinase genes. Chitinases play critical roles in multiple developmental stages of filamentous fungi, including sporulation, spore germination, hyphal growth, and hyphal autolysis. In *S. cerevisiae*, the disruption of the chitinase gene *CTS2* results in abnormal spore wall biosynthesis and failure to form mature asci [57]. *Rhizopus oligosporus* chitinase Chi3 loosens the cell wall at the hyphal tip, enabling turgor pressure to extend the hypha at the apex [58]. In *M. oryzae*, the deletion of the chitinase-encoding gene *Chi1* leads to reduced hyphal growth [51]. Similarly, in *Neurospora crassa*, the deletion of a chitinase gene disrupts hyphal cell wall remodeling [48]. Notably, while *chi18C* was transcriptionally downregulated in the con7-B mutant, it is known that in *A. nidulans*, the deletion of its homolog *chiA* gene leads to decreased spore germination and lower hyphal growth rates [59].

*Po*Con7 was also found to regulate the expression of CWDE genes, which was an unexpected result. We believe that the regulation of CWDE gene expression by *Po*Con7 is not a direct regulation, but rather a cascading indirect effect. The propose supported by the observed upregulation of the major activator ClrB, XlnR, and the downregulation of the repressor Ace1, both known to enhance CWDE production [45,46,47]. In fact, the deletion of *ace1* has been shown to increase *xlnR* transcription [60]. In the *xlnR* deletion strain, the transcript levels of *clrB* significantly decreased [61]. Interestingly, the negative regulator CreA was also upregulated, adding complexity to the regulatory landscape. However, the DAP-seq data did not identify significant peaks in the promoters of the TF genes *clrB*, *xlnR*, *ace1*, or *creA*. This suggests that *Po*Con7 does not directly regulate these TFs by binding to their promoters. Therefore, the proposed enhancement of (hemi)cellulase gene expression likely occurs through an indirect mechanism. Together, *Po*Con7 and these TFs, through their genetic relationships, dosage effects, and competitive interactions, form an intricate network that fine-tunes CWDE gene expression.

Similar to the phenotype observed in *Po*Con7-disrupted mutants, Snf1 orthologs in many filamentous fungi are key regulators of normal fungal growth and development as well as of chitinase gene expression [19,20,21,62]. Although the TF CreA, which is the fungal homolog of yeast Mig1 and a primary Snf1 target [18], was the anticipated interactor of *Po*Snf1, our TAP-MS analysis identified *Po*Con7 as the associated factor. Notably, CreA, a factor whose disruption/deletion/mutation upregulates CWDEs [63,64], and *Po*Con7 are involved in CWDE gene regulation, linking the Snf1 kinase pathway to CWDE expression control. However, CreA and *Po*Con7 exhibit a fundamental difference in their regulatory mechanisms. CreA displays dynamic nucleocytoplasmic trafficking mediated by glucose concentration [65]. In contrast, *Po*Con7 maintains constitutive nuclear localization, whether under glucose or cellulose signal conditions.

Y2H showed that *Po*Con7 directly interacts with *Po*Gal83, but not with the core catalytic subunits *Po*Snf1 or *Po*Snf4. As an adaptor protein, the ASC domain of *Po*Gal83 can simultaneously bind Snf4 and substrate proteins [15,66]. These findings support a model in which *Po*Con7 is indirectly linked to the SNF complex through *Po*Gal83, forming a transient regulatory module (*Po*Snf1–Gal83–Con7). As a target of Snf1, the phosphorylation status of CreA is known to modulate its transcriptional repressor activity [67]. The *Po*Con7 protein was analyzed for potential phosphorylation sites using NetPhos-3.1 (https://services.healthtech.dtu.dk/services/NetPhos-3.1/, accessed on 10 June 2024). Several residues, S244, Y38, S52, S108, T166, and S274, showed high phosphorylation potential, with prediction scores above 0.9 (Supplementary Spreadsheet S10). As these sites are predicted computationally, their actual phosphorylation status and functional relevance require further experimental validation.

## 4. Materials and Methods

### 4.1. Fungal Strains and Culture Conditions

The wild-type (WT) strain of *P. oxalicum* 114–2 (CGMCC 5302) served as the parent strain for all experiments. Both the WT and its mutants were cultured on agar plates supplemented with 10% wheat bran extract and incubated at 30 °C for five days to allow for conidiation. To assess mycelial development, the various strains were grown in 1× Vogel’s minimal medium (VMM) (50 × Vogel’s salt: 125.0 g Na_3_Citrate·2H_2_O, 250.0 g KH_2_PO_4_, 100.0 g NH_4_NO_3_, 10.0 g MgSO_4_·7H_2_O, 5.0 g CaCl_2_·2H_2_O, 0.25 mg biotin, 0.25 g citric acid, 0.25 g ZnSO_4_·7H_2_O, 0.05 g Fe(NH_4_)_2_(SO4)_2_·6H_2_O, 12.5 mg CuSO_4_·5H_2_O, 2.5 mg MnSO_4_·H_2_O, 2.5 mg H_3_BO_3_, 2.5 mg Na_2_MoO_4_·2H_2_O, and 1 L of water) [68] at 30 °C, plus 2% glucose (VMMG), 2% glycerol (VMMGl), or 2% sucrose (VMMS) as the sole carbon source. For solid-phase cultivation on plates, 1.5% agar was added to the VMMG, VMMGly, or VMMS.

### 4.2. Phylogenetic Analysis and Domain Architecture Analysis

The amino acid sequences of Con7 homologs of different species were obtained from NCBI database. We used the neighbor-joining method in Clustal X 1.83 [69] and MEGA 7.0 [70] to make multiple sequence alignments and draw physiological trees. Protein domain analysis was performed with SMART [71] and Pfam3 [72] databases. Domain architecture patterns were created in proportion to the corresponding protein sequences.

### 4.3. Construction of Different Mutants

All primers used in this study are listed in Supplementary Spreadsheet S11.

The Snf1-TAP strain, carrying a C-terminally FLAG-HA-tagged *Po*Snf1, was generated and validated as illustrated in Supplementary Figure S4A–C. To construct this strain, the 5′-upstream and 3′-downstream homologous arms flanking the *Po*Snf1 gene were amplified from the WT *P. oxalicum* genomic DNA using the primer pairs Snf1-TAP-UF/Snf1-TAP-UR and Snf1-DF/Snf1-DR, respectively. Primer pairs hph-F/hph-R were used to amplify the hygromycin B resistance gene (*hph*) from the plasmid Psilent1 [73]. Subsequently, the *hph* fragment, along with the 5′-upstream and 3′-downstream regions of the *Posnf1* gene, were fused via fusion PCR. The PCR product was further amplified using nested primers Snf1-TAP-CSF/Snf1-CSR and subsequently was transformed into the wild-type strain to generate the Snf1-TAP mutant. Correct integration of the FLAG-HA tag was confirmed by DNA sequencing.

The Con7-GFP strain (the protein *Po*Con7 fused with a green fluorescent protein (GFP)), was constructed, as shown in Supplementary Figure S5A,B. Briefly, the 5′-upstream and 3′-downstream homologous arms of the *Pocon7* gene were amplified from *P. oxalicum* WT genomic DNA using primers Con7-GFP-UF/Con7-GFP-UR and Con7-GFP-DF/Con7-GFP-DR. The *gfp* and *hph* marker genes were amplified from plasmids pEGFP and Psilent1 with primer pairs gfp-F/gfp-R and hph-F/hph-R, respectively. The four fragments (the two homologous arms of the *Pocon7* gene, *gfp*, and *hph*) were then fused by fusion PCR. The PCR product was amplified with nested primers Con7-GFP-CSF/Con7-GFP-CSR and transformed into the WT strain to generate the Con7-GFP mutant.

The truncated mutant con7-B (the gene *Pocon7* was partially disrupted) was constructed, as shown in Supplementary Figure S5C,D. To construct this strain, the 5′-upstream and 3′-downstream homologous arms of the *Pocon7* gene were amplified from *P. oxalicum* WT genomic DNA using primers con7-B-UF/con7-B-UR and con7-B-DF/con7-B-DR. Primer pairs hph-F/hph-R were used to amplify the marker gene *hph*. Subsequently, the *hph* fragment, along with the 5′-upstream and 3′-downstream regions of the *Pocon7* gene, were fused via fusion PCR. The PCR product was further amplified using nested primers con7-B-CSF/con7-B-CSR and subsequently was transformed into the WT strain to generate the con7-B mutant.

### 4.4. Fungal Colony and Microscopic Observation

Fresh spore suspensions (10^7^/mL) from different strains were prepared and spotted (2 μL) onto VMMG, VMMGly, or VMMS, or onto potato dextrose agar (PDA). Plates were incubated at 30 °C for 4 days to assess colonial growth.

For observing hyphae in the Con7-GFP strain, 100 μL of fresh spore suspensions was spread on VMMG or VMMC (VMM with 2% ball-milled cellulose) agar inserting sterile coverslips. After 24 h at 30 °C, the coverslips were removed, and the mycelia were stained with 1 µg/mL Hoechst 33,342 for 15 min in the dark for nuclear staining. Samples were imaged using a ZEISS LSM900 laser scanning confocal microscope (Zeiss, Jena, Germany) with excitation values at 488 nm (GFP) and 405 nm (nuclei).

### 4.5. Tandem Affinity Purification and Mass Spectrometry

Fresh conidial suspensions of *P. oxalicum* WT and Snf1-TAP strains were cultured in 2 L of VMMG liquid medium at 30 °C with shaking at 200 rpm for 24 h. The harvested mycelia were washed twice with 0.96% NaCl (*w*/*v*) containing 1% DMSO and 1 mM PMSF, then ground under liquid nitrogen. The resulting powder was resuspended in 15 mL of protein lysis buffer (containing 9 g/L NaCl, 1 M Tris-HCl pH 7.5, 100 mL/L glycerol, 10 mL/L NP-40, and 0.05% Protease Inhibitor Cocktail (MedChemExpress, Shanghai, China)) and incubated on ice for 10 min. After centrifugation at 10,000 rpm and 4 °C for 30 min, the supernatant was collected.

For the first affinity purification step, the supernatant was incubated with ANTI-FLAG M2 Affinity Resin (Smart-Lifesciences, Changzhou, China) overnight at 4 °C with gentle rotation. Bound proteins were eluted by competition with 500 µL of 3×FLAG peptide (150 ng/µL). The eluate was then subjected to a second affinity purification using ANTI-HA Resin (Smart-Lifesciences, China) under the same incubation conditions. Final elution was performed using 80 µL of 8 M urea to obtain the final protein eluate.

The final eluate was divided for three parts: separation by 12.5% SDS-PAGE followed by silver staining; Western blot using an anti-HA antibody (ABclonal, Wuhan, China); and LC-MS/MS (APT, Shanghai, China) for protein identification. Relative protein abundance was estimated using the emPAI (exponentially modified Protein Abundance Index) method [31], where emPAI = 10^PAI^ − 1, and PAI represents the ratio of experimentally observed to theoretically observable tryptic peptides (N_observed_/N_observable_) for each protein.

### 4.6. Total RNA Extraction and Gene Expression Analysis by RT-qPCR

Fresh spore suspensions from different strains were incubated in VMMG liquid medium at 30 °C for 24 h. Subsequently, 0.3 g of filtered hyphae was transferred to 50 mL of fresh VMMG and VMMC and cultured at 30 °C with shaking at 200 rpm. After 24 h, the mycelia were collected by centrifugation and ground in liquid nitrogen. Total RNA was extracted from 100 mg of the ground powder using TRIzol reagent (TaKaRa, Kyoto, Japan) according to the manufacturer’s instructions. cDNA was synthesized with the PrimeScript RT Reagent Kit with gDNA Eraser (TaKaRa, Japan). Quantitative PCR was performed using a LightCycler 480 system with software version 4.0 (Roche, Basel, Germany) with specific primers for *PobrlA*, *Poabr2*, and *Poactin* (see Supplementary Spreadsheet S11). The *Poactin* gene (PDE_01092) was used as the internal control. Relative expression was calculated as the ratio of the target gene copy number to that of actin. All experiments were conducted with three biological replicates, and statistical significance was defined as *p* ≤ 0.05.

### 4.7. Transcriptome Analysis and GO Analysis

Strains were cultured as described in the “Real-time quantitative PCR” section. After 24 h of growth in VMMC, fresh mycelia of the WT and con7-B strains were collected, ground in liquid nitrogen, and total RNA was extracted using RNAiso Plus reagent (Takara, Kyoto, Japan). To remove genomic DNA, the RNA was treated with 10 U of DNase I at 37 °C for 30 min. mRNA quality was verified based on the following criteria: OD_260_/OD_280_ between 1.8–2.2, OD_260_/OD_230_ greater than 1.5, and an RNA integrity number (RIN) above 8.0. Transcriptome sequencing was conducted on the BGISEQ-500 platform at the Beijing Genomics Institute (BGI, Shenzhen, China). Sample saturation analysis was performed to confirm suitability for omics studies.

Clean reads for each gene were normalized using the fragments per kilobase transcript per million mapped reads (FPKM) for differential expression analysis. Significantly differentially expressed genes were identified with the thresholds |log_2_(FoldChange)| ≥ 1 and Q-value < 0.05. Functional enrichment and GO annotation were carried out with ShinyGO v0.82 (FDR ≤ 0.05), and cluster analysis was performed using Genesis 1.0 software [74].

### 4.8. Yeast Two-Hybrid Assay

For yeast two-hybrid (Y2H) assays, truncated versions of *Po*Con7 CDS (avoiding the DNA-binding domain; amino acids 1–192 and 225–342) were amplified using primers con7-BDF/con7-BDR. The products were cloned into the plasmid pGADT7 and transformed into *S. cerevisiae* Y187. Similarly, the CDS sequences of *Po*Gal83, *Po*Snf4, and *Po*Snf1 were amplified with specific primer Gal83-BDF/Gal83-BDR, Snf4-BDF/Snf4-BDR and Snf1-BDF/Snf1-BDR, and cloned into pGBKT7, which was then transformed into *S. cerevisiae* Y2HGold. Protein–protein interactions were assessed by growing the Y2H strains on QSD (SD-Leu/-Trp/-His/-Ade) and QSD/X/Aba (SD-Leu/−Trp/-His/−Ade/x-α-gal/Aba) agar plates.

### 4.9. Protein Expression and Purification

The codon-optimized coding sequence of *Po*Con7 was synthesized by GenScript Biotech Corporation (Nanjing, China) for heterologous expression in *E. coli*. Following digestion with *Sal*I and *Xho*I, the fragment was ligated into similarly digested pET28b(+). The resulting recombinant plasmid was transformed into *E. coli* BL21 (DE3) for protein expression. The transformed cells were cultured in LB medium supplemented with 30 mg/mL kanamycin at 37 °C until OD_600_ reached a range of 0.6–0.8. Expression was induced with 0.4 mM isopropyl-β-D-1-thiogalactopyranoside (IPTG) at 18 °C for 16 h. Cells were harvested by centrifugation (14,000 *g*, 4 °C, 15 min), washed with PBS, and resuspended in lysis buffer (50 mM Tris, 500 mM NaCl, pH 7.0). After sonication on ice, the lysate was centrifuged under the same conditions. The supernatant was filtered (0.22 μm) and loaded onto a 1 mL HisTraTM HP column (Smart-Lifesciences, China) pre-equilibrated with buffer A (50 mM Tris, 500 mM NaCl, pH 7.0). The column was washed with buffer A, followed by elution using buffer A containing 500 mM imidazole. The eluted protein was dialyzed against 50 mM Tris, 150 mM NaCl (pH 7.0). Protein concentration was determined using a Modified Bradford Protein Assay Kit (Sango Biotech, Shanghai, China) with BSA as the standard.

### 4.10. DNA Affinity Purification Sequencing Assays

The DNA Affinity Purification Sequencing (DAP-seq) assay was conducted based on a previously described method [75] with minor modifications. Briefly, fresh spore suspensions of the *P. oxalicum* WT strain were cultured in VMMG liquid medium at 30 °C for 24 h. The mycelia were ground in liquid nitrogen, and genomic DNA was isolated via phenol extraction and ethanol precipitation. The DNA was then broken into approximately 500 bp fragments by sonication at 35% power output.

The recombinant transcription factor protein was incubated with the fragmented genomic DNA library. Complexes of the target protein and bound DNA were isolated using His-labeled protein purified agarose magnetic beads (Beyotime, China), followed by digestion with Proteinase K (Vazyme, China) to release the DNA. The purified DNA fragments were used for library construction and sequenced on the DNBSEQ platform (BGI, Shenzhen, China).

Raw sequencing data from the DAP-seq were filtered to obtain clean reads, which were aligned to the reference genome using Bowtie2. The aligned results were visualized, and peak calling was performed with MACS2 to identify transcription factor binding sites. Genomic annotation of peaks was conducted to determine their distribution across functional elements. De novo motif analysis within the peak regions was carried out using the MEME suite (https://meme-suite.org/meme/tools/meme 5.5.9, accessed on 10 June 2024).

### 4.11. ChIP-qPCR Analysis

Chromatin immunoprecipitation (ChIP) assays were carried out with modifications based on established protocols [76,77]. In brief, hyphae grown in VMMG medium were cross-linked with 1% formaldehyde for 10 min, and fixation was stopped by adding 125 mM of glycine for 5 min. The harvested hyphae were ground in liquid nitrogen and lysed in lysis buffer (50 mM HEPES pH 7.5, 150 mM NaCl, 1 mM EDTA, 0.5% Triton X-100, 0.1% sodium deoxycholate, 0.1% SDS, 1 mM PMSF, and 0.1% protease inhibitor cocktail). After centrifugation, the collected chromatin was sonicated to an average size of approximately 500 bp at 35% power output.

Immunoprecipitation (IP) was conducted using an anti-HA antibody (Proteintech, Chicago, IL, USA) and protein A/G magnetic beads (Thermo Fisher Scientific, Waltham, MA, USA), with 1 mg of chromatin used per reaction. Both the obtained IP products and 0.1 mg input chromatin DNA (without IP) of each sample were treated with RNase, followed by a reversal of cross-links through heating and proteinase K digestion. Then, IP DNA and input DNA were purified by phenol extraction and ethanol precipitation. Quantitative PCR was performed on a LightCycler 480 instrument (Roche, Mannheim, Germany) with primers listed in Supplementary S11. The relative enrichment of target DNA was calculated using the Input% method as follows (where Ct = the number of qPCR cycles required to reach the threshold):

ChIP efficiency = 2^−ΔCt^ × 100%

ΔCt = Ct_IP_ − (Ct_Input_ − log_2_10)

Three biological triplicates were performed for all strain samples.

## 5. Conclusions

The transcription factor *Po*Con7 is an essential regulator of fungal viability and a novel target of the SNF complex, specifically interacting with the Gal83 subunit. It directly controls chitinase expression by binding to the 5′-TATTWTTAT-3′ motif in the promoters of chitinase genes. *Po*Con7 governs the expression of a broad range of CWDE genes, including those encoding cellulases and hemicellulases, demonstrating its extensive regulatory function. These findings show the key role of *Po*Con7 in cell wall metabolism and its potential as a target for optimizing enzyme production in white biotechnology.

## Data Availability

Raw MS/MS data for TAP-MS are deposited in iProX (https://www.iprox.cn) under accession codes PXD068340. The raw data of transcriptome sequencing have been deposited in NCBI’s Gene Expression Omnibus database under the accession numbers GSE308026.

## Supplementary Materials

The following supporting information can be downloaded at: https://www.mdpi.com/article/10.3390/ijms27010333/s1.
