# Supplementary figures and images for: The Transcription Factor PoCon7 Is Essential for Fungal Viability and Regulates Chitinase Gene Expression in Penicillium oxalicum
Source: Int J Mol Sci. 2025 Dec 28;27(1):333. doi: 10.3390/ijms27010333 (PMC12785316; doi:10.3390/ijms27010333)

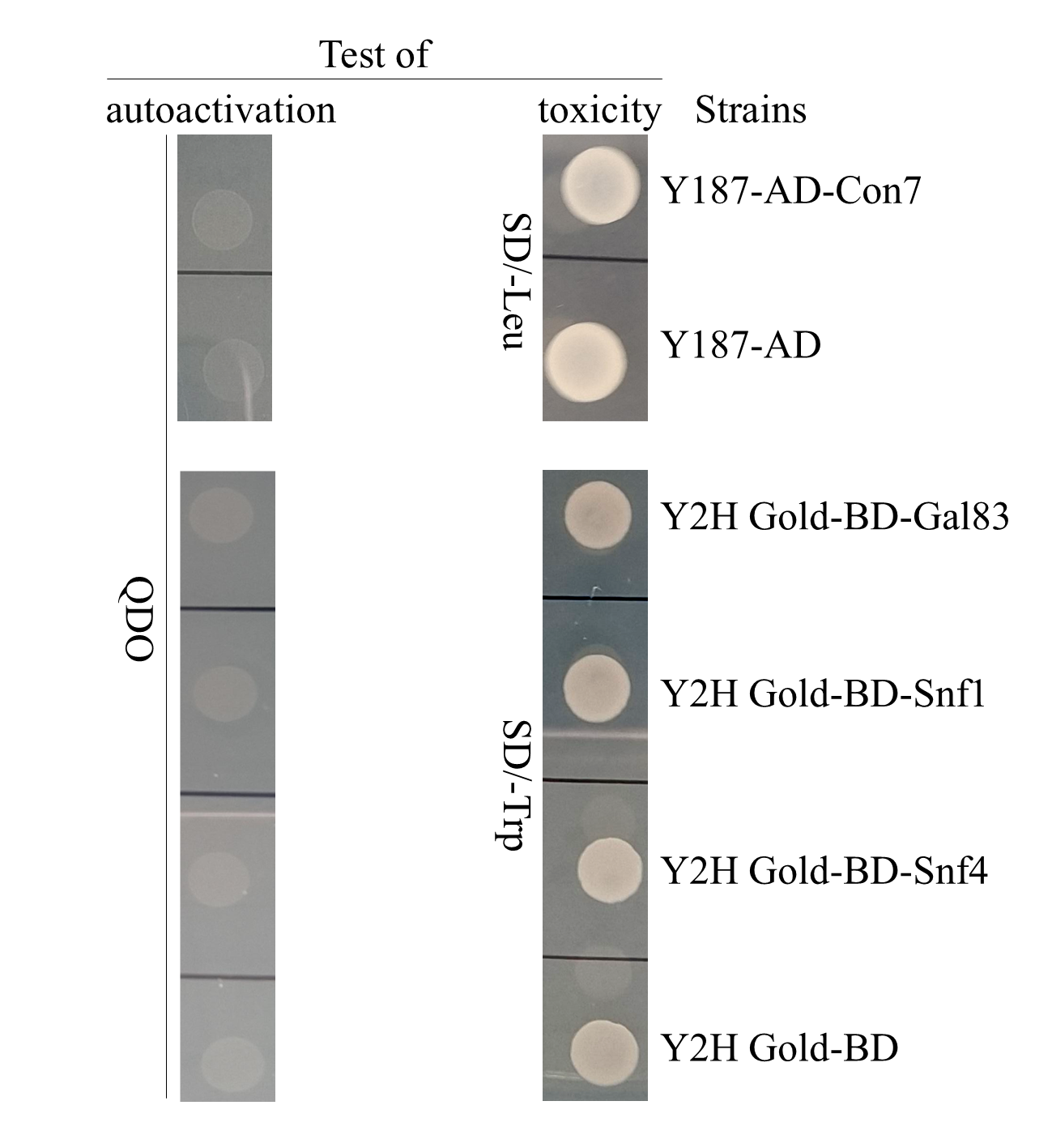

Supplement: Supplementary file 1 [file ijms-27-00333-s001.zip › FigS1.tif]

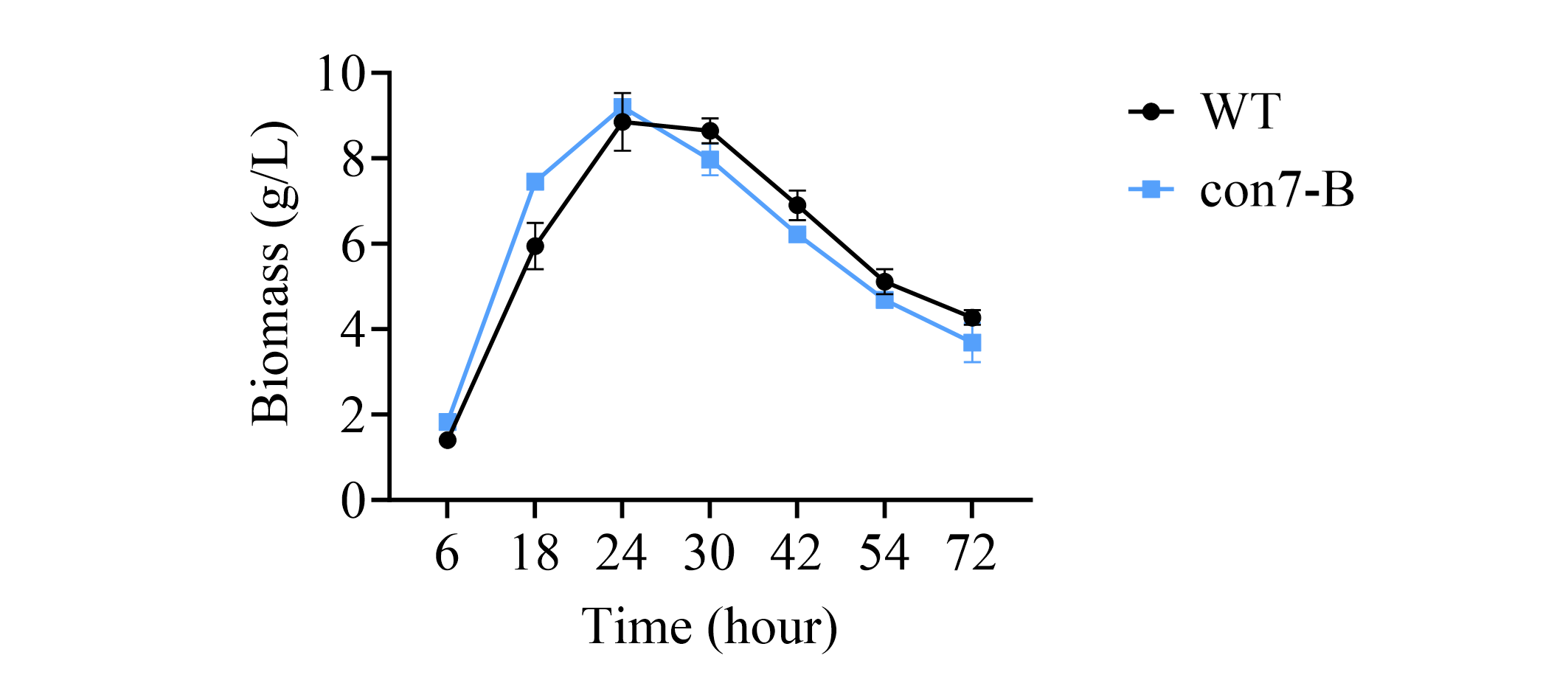

Supplement: Supplementary file 1 [file ijms-27-00333-s001.zip › FigS2.tif]

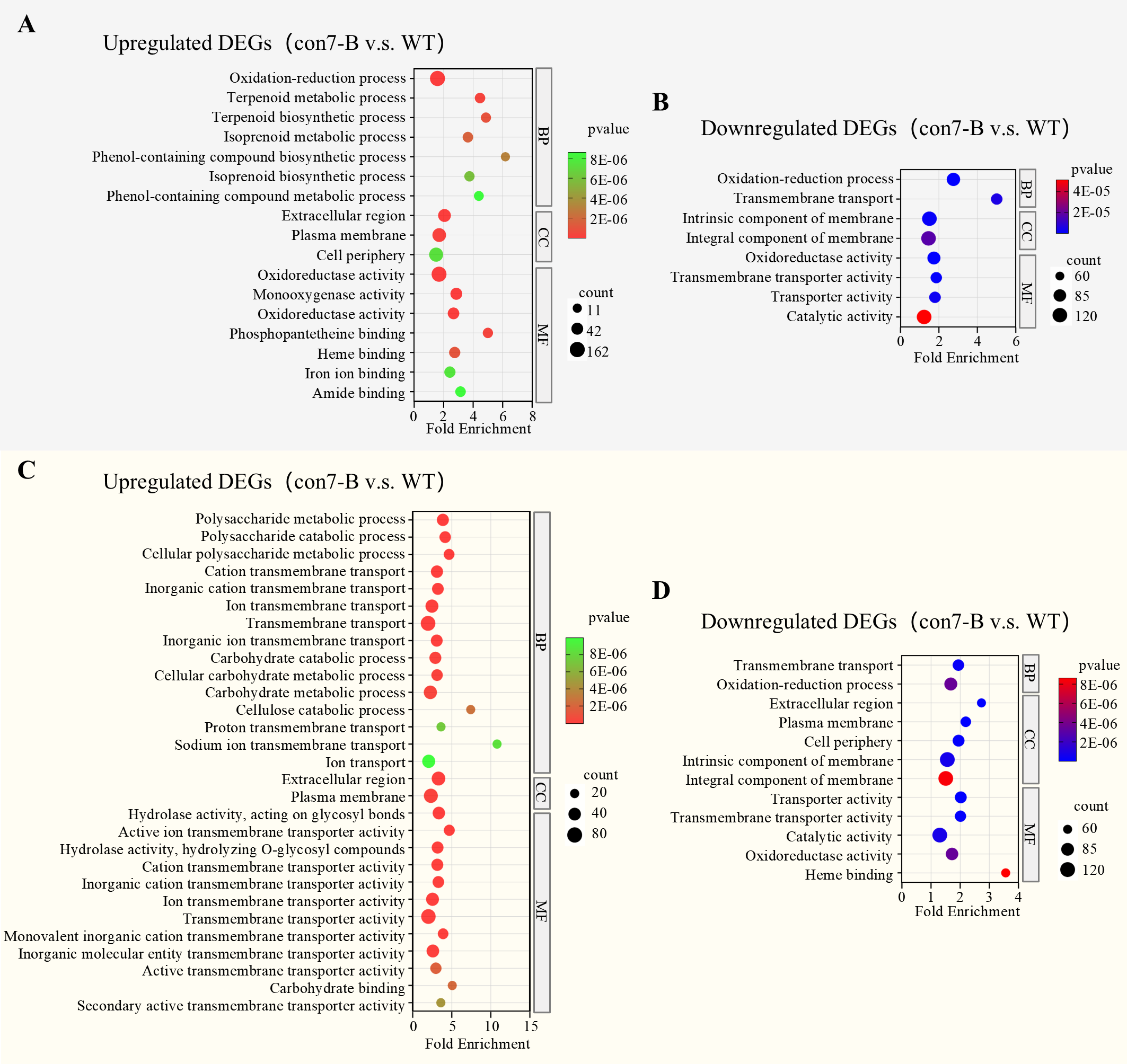

Supplement: Supplementary file 1 [file ijms-27-00333-s001.zip › FigS3.tif]

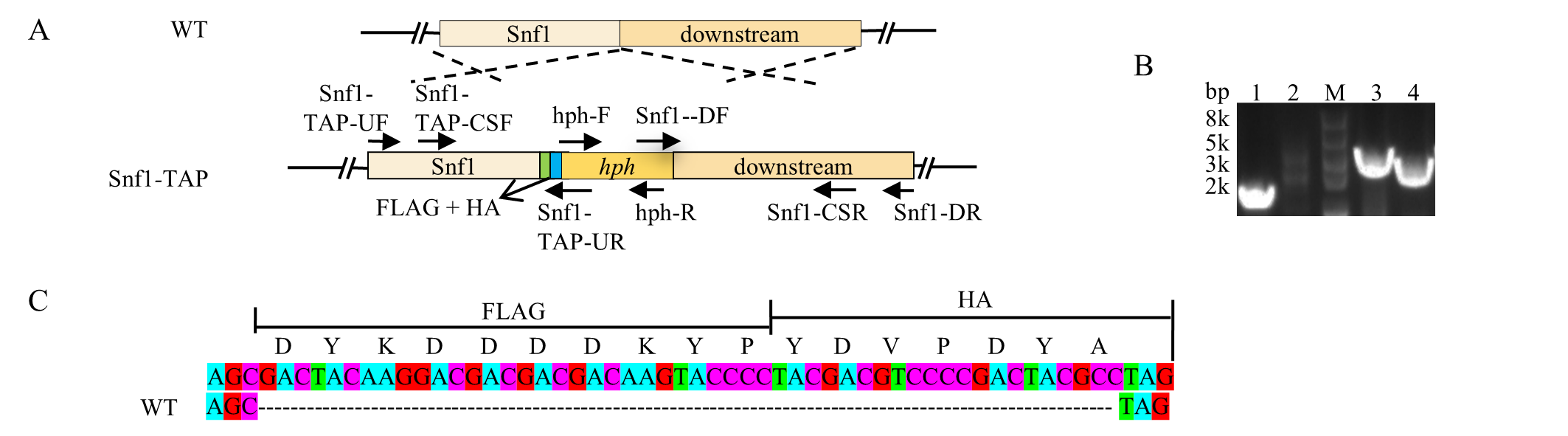

Supplement: Supplementary file 1 [file ijms-27-00333-s001.zip › FigS4 .tif]

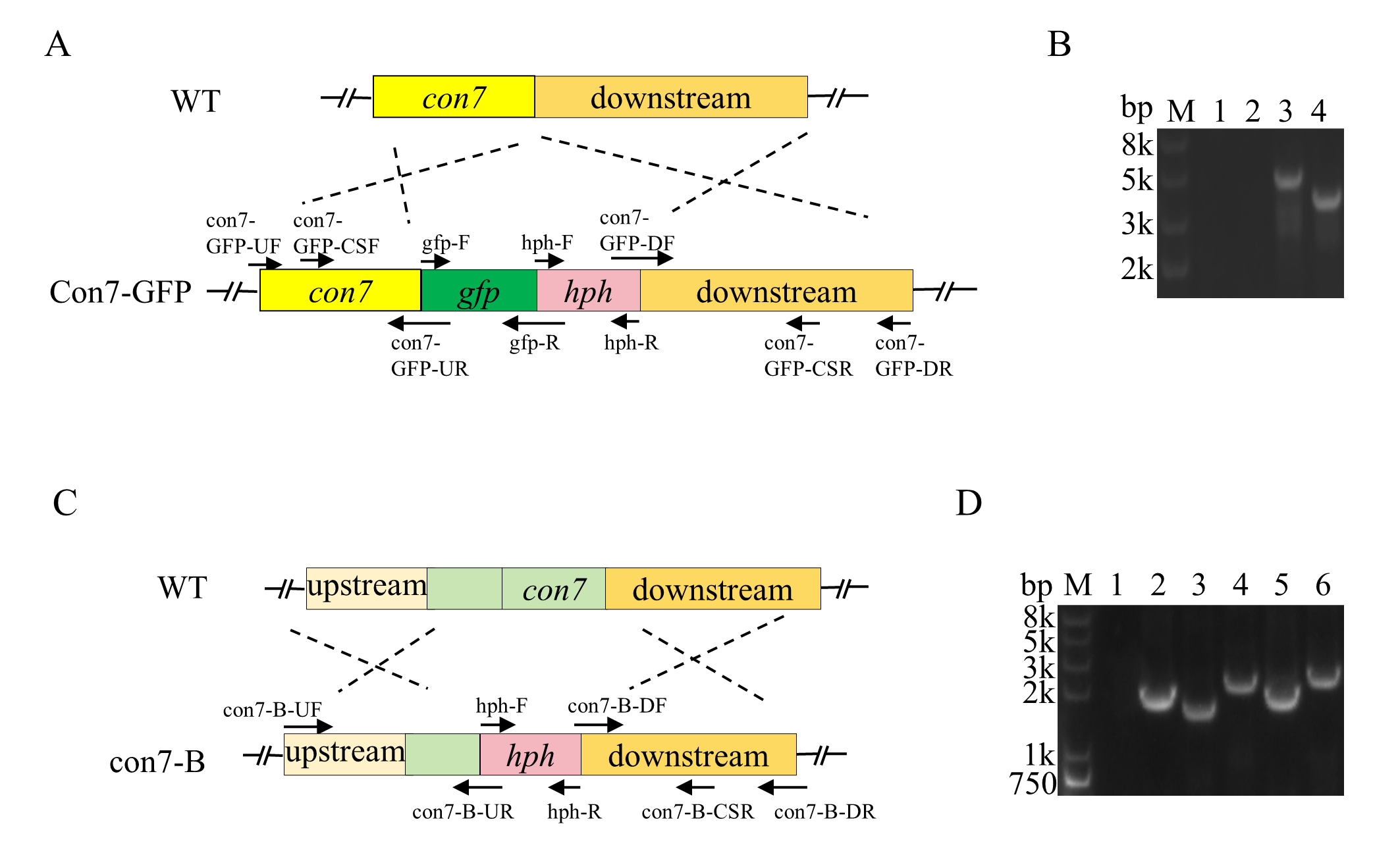

Supplement: Supplementary file 1 [file ijms-27-00333-s001.zip › FigS5.tif]
